# Differential responses of epithelial cells from urinary and biliary tract to eggs of *Schistosoma haematobium* and *S*. *mansoni*

**DOI:** 10.1038/s41598-019-46917-y

**Published:** 2019-07-24

**Authors:** Rafael Nacif-Pimenta, Alessandra da Silva Orfanó, Ilana A. Mosley, Shannon E. Karinshak, Kenji Ishida, Victoria H. Mann, Paulo Marcos Zech Coelho, José M. Correia da Costa, Michael H. Hsieh, Paul J. Brindley, Gabriel Rinaldi

**Affiliations:** 1 Department of Microbiology, Immunology & Tropical Medicine, and Research Center for the Neglected Diseases of Poverty, School of Medicine and Health Sciences, George Washington University, Washington DC, USA; 2 Laboratório de Esquistossomose, Instituto René Rachou, Fundação Oswaldo Cruz - FIOCRUZ, Belo Horizonte, Minas Gerais Brazil; 3 Laboratório de Entomologia Médica, Instituto René Rachou, Fundação Oswaldo Cruz - FIOCRUZ, Belo Horizonte, Minas Gerais Brazil; 4 Biomedical Research Institute, Rockville, Maryland USA; 5 Department of Infectious Diseases, R&D Unit, INSA-National Health Institute Dr. Ricardo Jorge, Porto, Portugal; 6 Center for the Study of Animal Science, ICETA, University of Porto, Porto, Portugal; 7 Department of Urology, School of Medicine and Health Sciences, George Washington University, Washington DC, USA; 8 Children’s National Health System, Washington DC, USA; 9 Present Address: Wellcome Sanger Institute, Wellcome Genome Campus, Hinxton, UK

**Keywords:** Parasite host response, Bladder cancer, Oncogenes, Bladder cancer

## Abstract

Chronic urogenital schistosomiasis can lead to squamous cell carcinoma of the bladder. The International Agency for Research on Cancer classifies the infection with *S*. *haematobium* as a group 1 carcinogen, a definitive cause of cancer. By contrast, hepatointestinal schistosomiasis due to the chronic infection with *S*. *mansoni* or *S*. *japonicum* associated with liver periportal fibrosis, does not apparently lead to malignancy. The effects of culturing human epithelial cells, HCV29, established from normal urothelium, and H69, established from cholangiocytes, in the presence of *S*. *haematobium* or *S*. *mansoni* eggs were investigated. Cell growth of cells co-cultured with schistosome eggs was monitored in real time, and gene expression analysis of oncogenesis, epithelial to mesenchymal transition and apoptosis pathways was undertaken. Schistosome eggs promoted proliferation of the urothelial cells but inhibited growth of cholangiocytes. In addition, the tumor suppressor P53 pathway was significantly downregulated when exposed to schistosome eggs, and downregulation of estrogen receptor was predicted in urothelial cells exposed only to *S*. *haematobium* eggs. Overall, cell proliferative responses were influenced by both the tissue origin of the epithelial cells and the schistosome species.

## Introduction

The blood flukes *Schistosoma japonicum* and *S*. *mansoni* are agents of hepatointestinal  schistosomiasis in East Asia, Africa, northeastern South America and the Caribbean, whereas *S*. *haematobium* causing urogenital schistosomiasis (UGS) is present through Africa and the Middle East. It is estimated that 4.5 to 70 million disability adjusted life years (DALYs) are lost due to schistosomiasis^1^, and of >100 million cases of UGS in sub-Saharan Africa, 70 million show hematuria, 18 million major bladder pathology, and 10 million hydronephrosis that would eventually lead to kidney damage^2,3^. Many of the eggs of *S*. *haematobium* become trapped in host tissues, in particular urogenital organs, leading to inflammation and eventually squamous cell carcinoma of the bladder (SCC)^4^. Accordingly, and based on convincing epidemiological and pathophysiological findings, UGS has been classified as group 1 carcinogen by the International Agency for Research on Cancer^5^, although the cellular and molecular mechanisms underlying this infection-related carcinogenic process remain unclear. Women with UGS may suffer from female genital schistosomiasis (FGS)^6^ as consequence of the schistosome egg deposition in the uterus, cervix, vagina and vulva. Moreover, FGS has been associated with female infertility^7^ and increased susceptibility to HIV^8^.

Schistosome eggs in the bladder wall release metabolites, presumably to facilitate the egress to the lumen and subsequently to the external environment to propagate the transmission cycle. Mass spectrometric analysis of urine during UGS has revealed estrogen-like metabolites, catechol estrogen quinones (CEQ)-DNA-adducts and novel metabolites derived from 8-oxo-7, 8-dihydro-2′- deoxyguanosine (8-oxodG)^9^ representing potential bladder carcinogens that may directly damage the DNA, leading to somatic mutations in oncogenes and tumor suppressors^10,11^. By contrast, *S*. *mansoni* dwells in the mesenteric vessels releasing eggs that embolize within the presinusoidal capillary beds of the liver, inducing periportal fibrosis and portal hypertension. Hepatointestinal schistosomiasis does not apparently lead to cell malignant transformation in these organs^5^.

Epithelial carcinomas are typically classified as conventional and nonconventional carcinomas^12^; 90% of epithelial carcinomas are of the conventional type and result from either papillary or flat *in situ* lesions, while nonconventional carcinomas include SCC, adenocarcinoma, and small cell carcinoma. SCC of the bladder is characterized by invasive cells containing desmosomes with keratin formation^12^. Research of UGS-induced bladder cancer is challenging due to the absence of laboratory animal models that mirror the human disease; in rodent models the vast majority of *S*. *haematobium* adult worms reside in the mesenteric veins. Recently, a mouse model was developed by injecting eggs of *S*. *haematobium* into the bladder wall of mice provoking egg-associated pathogenesis similar to the human condition^13,14^. In addition, premalignant lessons associated with epithelial to mesenchymal (EMT)-like profiles occurred following co-administration of nitrosamine in this model^15^.

In this study, responses of urothelium (HCV29 cells) and bile duct epithelium (H69 cells) to eggs of either *S*. *haematobium* or *S*. *mansoni* were investigated. Cells were cultured in the presence of schistosome eggs, and cellular proliferation monitored in real time using the xCELLigence system^16^. Increased proliferation of urothelial cells was evident when exposed to schistosome eggs, in particular to *S*. *haematobium* eggs. On the other hand, eggs of both schistosome species induced cell death of cholangiocytes. These phenotypic effects were associated with dysregulation of genes involved in oncogenesis, epithelial-mesenchymal transition and apoptosis pathways. Future studies to decipher cellular and/or molecular mechanisms underlying the association between UGS and bladder cancer will contribute to the discovery of new interventions for this neglected tropical disease-related cancer.

## Results

### Schistosome eggs promoted growth of urothelial cells but inhibited cholangiocytes

A real-time cell proliferation assay was employed to measure the effect of co-culturing schistosome eggs with two informative human epithelial cell lines. Although we have previously studied human cholangiocyte cells H69 employing the xCELLigence Real Time Cell Assay^17^, we had not quantified the proliferation of the human urothelial cell line HCV29 using this approach. It is critical to set up *a priori* the conditions for cell proliferation analysis for every new cell line in the laboratory mediated xCELLigence, even though the same cell line has been already analyzed by others^18^. Therefore, cell titration experiments were undertaken in order to establish tractable assay parameters, including seeding cell density (Fig. S1). Notably, regardless of the initial number of seeded cells, all the tested conditions showed a delay of at least ~24 hours before the cell index (CI) started to increase, with 20,000 cells per well being the condition that reached a CI of ~3.0 within ~20 hours after it started to increase. Therefore, a seeding cell density of 20,000 cells per well was used in subsequent analyses.

**Figure 1:**
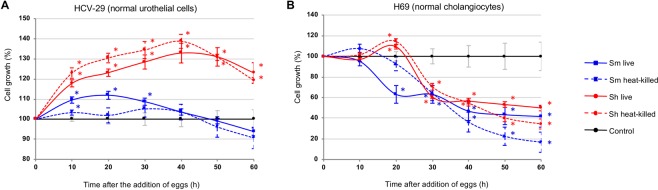
Proliferation of urothelial cells and cholangiocytes in response to schistosome eggs, monitored in real time using the xCELLigence system. Non-contact co-culture of *Schistosoma mansoni* and *S*. *haematobium* eggs with HCV29 cells (Panel A) or H69 cells (Panel B) over time after the addition of the eggs. Cell growth is expressed as percentage of the Normalized Cell Index of cells co-cultured with eggs compared with control cells (control cell growth rate = 100%). All curves represent the averages of at least three technical replicates for the experiment and standard deviations are shown as error bars at each data point. Blue and red asterisks indicate levels of significance (*P* ≤ 0.01) of growth ratios of cells cultured with *S*. *mansoni* and *S*. *haematobium* eggs, compared to control cells cultured in the absence of eggs, respectively. Sm live: *S*. *mansoni* live eggs; Sm heat-killed: heat-killed *S*. *mansoni* eggs; Sh live: *S*. *haematobium* live eggs; Sh heat-killed: heat-killed *S*. *haematobium* eggs.

HCV29 cells were seeded in E-plates, and cultured until the CI reached ~1.0. Subsequently, the cells were starved for two to four hours in 1/20 diluted medium before being co-cultured with schistosome eggs. Within ~10 hours following addition of the eggs, proliferation of the cells accelerated, in an egg-concentration dependent manner in comparison to cells cultured without schistosome eggs. Eggs of both *S*. *mansoni* and *S*. *haematobium* induced cell proliferation, although eggs of *S*. *haematobium* induced higher proliferation, i.e. ~30% versus ~10% by eggs of *S*. *haematobium* and *S*. *mansoni*, respectively (Figs 1A and S2A). Notably, heat-killed eggs showed similar effects to live eggs on HCV29 cell proliferation (Fig. 1A). Similarly, H69 cells were co-cultured in the presence of schistosome eggs. In contrast to HCV29, schistosome eggs (live or dead) inhibited cell proliferation of cholangiocytes (Figs 1B and S2B). Although the *S*. *haematobium* eggs briefly induced cell proliferation of H69 cells between ~15 to 20 h post-incubation, eggs of both species overall reduced cell growth by ≥30% (Fig. 1B). The cell proliferation inhibition of H69 cells was evident by 24 h post-incubation for both schistosome species.

To summarize, the effect of the eggs on cell proliferation depended mainly on the cell type, i.e. cholangiocytes or urothelial, rather than on the schistosome species; however, *S*. *haematobium* eggs induced more proliferation of HCV29 cells than *S*. *mansoni* eggs (Fig. 1A), and a brief increase of H69 cell proliferation before the cell inhibition was evident for both schistosome species. Surprisingly, this proliferative effect was independent of egg viability. However, a cell proliferation increment was also evident when HCV29 cells were exposed to excretory-secretory products (ES) previously collected from either *S*. *mansoni* or *S*. *haematobium* eggs (Fig. S3), strongly suggesting that the proliferative effect was not due to the presence of a ‘foreign body’ inducing a non-specific effect. Overall, the same trend was evident for both tested conditions, i.e. eggs and ES. When ES products collected from both schistosome egg species were employed in the assay the cell proliferation increased in a concentration-dependent manner. Evidently 10 μg/ml, the highest tested concentration, induced the strongest proliferation, thus the cells started to die sooner. Remarkably, this effect was more pronounced when *S*. *haematobium* was employed (Fig. S3). Further studies comparing the compositions of the *S*. *mansoni* and *S*. *haematobium* ES products may lead to a better understanding of these differences.

### Gene expression dysregulation was schistosome species- and contact time- dependent

**Figure 2:**
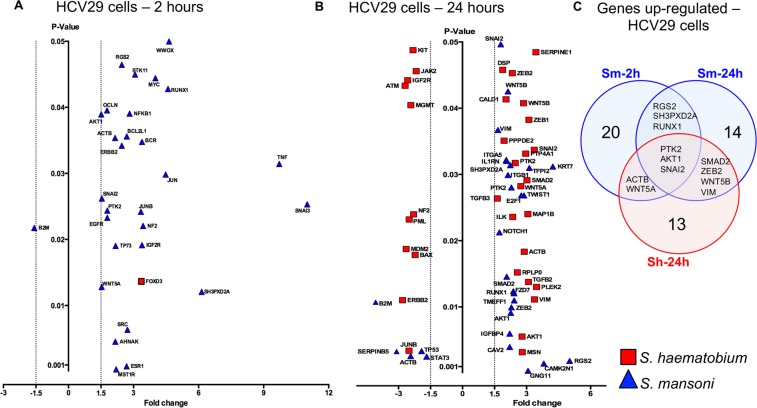
Schistosome eggs induced dysregulation of oncogenes, tumor suppressors and EMT-related genes in HCV29 cells over time. Volcano plots of HCV29 cell gene response to non-contact co-culture with *S*. *mansoni* or *S*. *haematobium* eggs as indicated, for 2 (Panel A) or 24 (Panel B) hours. Gene expression was measured using qPCR gene arrays designed to assess oncogenesis- and EMT- associated transcripts. Significantly dysregulated (*P* < 0.05) genes with >±1.5-fold-change, are shown. Panel C. Venn diagram compiled only with the HCV29 cell culture conditions that share differentially expressed genes (DEG) – only upregulated genes were shared among the 3 indicated conditions. (Table S2 includes the complete set of DEG indicated in the Venn diagram).

The effect of schistosome eggs on cell proliferation of HCV29 was evident within the first 10 hours after the incubation and persisted beyond 24 hours. Therefore, guided by the xCELLigence findings we decided to analyze the gene expression of cells at two time points post-incubation; cells exposed for 2 hours (‘early time point’) looking for early events in gene expression dysregulation, and cells exposed for 24 hours. Two gene qPCR arrays were employed to detect ‘early’ and ‘late’ molecular events that might be associated with high cell proliferation of the urothelial HCV29 cells co-cultured with schistosome eggs; (1) oncogenes and tumor suppressor gene array, and (2) epithelial to mesenchymal transition (EMT) gene array. The output raw data from these two gene arrays were combined in the final analysis. Whereas urothelial cells rapidly displayed gene dysregulation two hours after the addition of *S*. *mansoni* eggs, *S*. *haematobium* only induced the upregulation of the transcriptional repressor FOXD3 (Fig. 2A). At 2 hours, the early time point, *S*. *mansoni* eggs co-cultured with urothelial cells triggered the upregulation of 28 genes and downregulation of a single gene (B2M - a component of MHC class I molecules.) (Fig. 2A and Table S1). Tumor Necrosis Factor (TNF) and SNAI3 genes were the most upregulated genes in HCV29 cells co-cultured with *S*. *mansoni* eggs with fold changes of 9.72 and 11.01, respectively. Notably, the estrogen receptor 1 gene (ESR1) was upregulated 2.68-fold in HCV29 cells co-cultured with *S*. *mansoni* eggs at the ‘early’ time point.

Urothelial cells exposed to *S*. *mansoni* eggs for 24 h (‘late time point’) displayed 29 dysregulated genes (Fig. 2B and Table S1). Interestingly, at this late time point, *S*. *mansoni* eggs induced downregulation of *p53* tumor suppressor gene by 1.92-fold (Fig. 2B, shown as TP53, and Table S1). The expression of 7 genes dysregulated at the early time point remained altered at the late time point (ACTB, AKT1, B2M, RGS2, RUNX1, SH3PXD2A, SNAI2) (Fig. 2B and Table S1). Although at the early time point a single gene was upregulated, at the late time point 33 genes were dysregulated by *S*. *haematobium* eggs (Fig. 2B). Seven genes were dysregulated by both *S*. *mansoni* and *S*. *haematobium* eggs at the late time point (ACTB, AKT1, SMAD2, SNAI2, VIM, WNT5B, ZEB2) (Fig. 2B and Table S1). It is noteworthy that *S*. *mansoni* eggs upregulated 3.39 folds the expression of Insulin Like Growth Factor 2 Receptor (IGF2R) in urothelial cells at both time points examined, the same gene was 2.57-fold downregulated by *S*. *haematobium* (Fig. 2B and Table S1). Overall, 64 genes were dysregulated by *S*. *mansoni* and *S*. *haematobium* eggs when co-cultured with urothelial cells (Fig. 2B and Table S1). Cell culture conditions that displayed overlapped differentially expressed genes are shown in a Venn diagram (Fig. 2C). Surprisingly, relatively few upregulated genes were shared between early and late time points in HCV29 cells exposed to *S*. *mansoni* eggs, i.e. RGS2, SH3PXD2A, and RUNX1. Three genes, PTK2, AKT1, and SNAI2 were significantly upregulated in cells exposed to both schistosome species; *S*. *mansoni* eggs at both time points and *S*. *haematobium* eggs at the late time point. (Fig. 2C, and Table S2). No downregulated genes were shared among the different cell culture conditions for HCV29 cells (Table S2).

**Figure 3:**
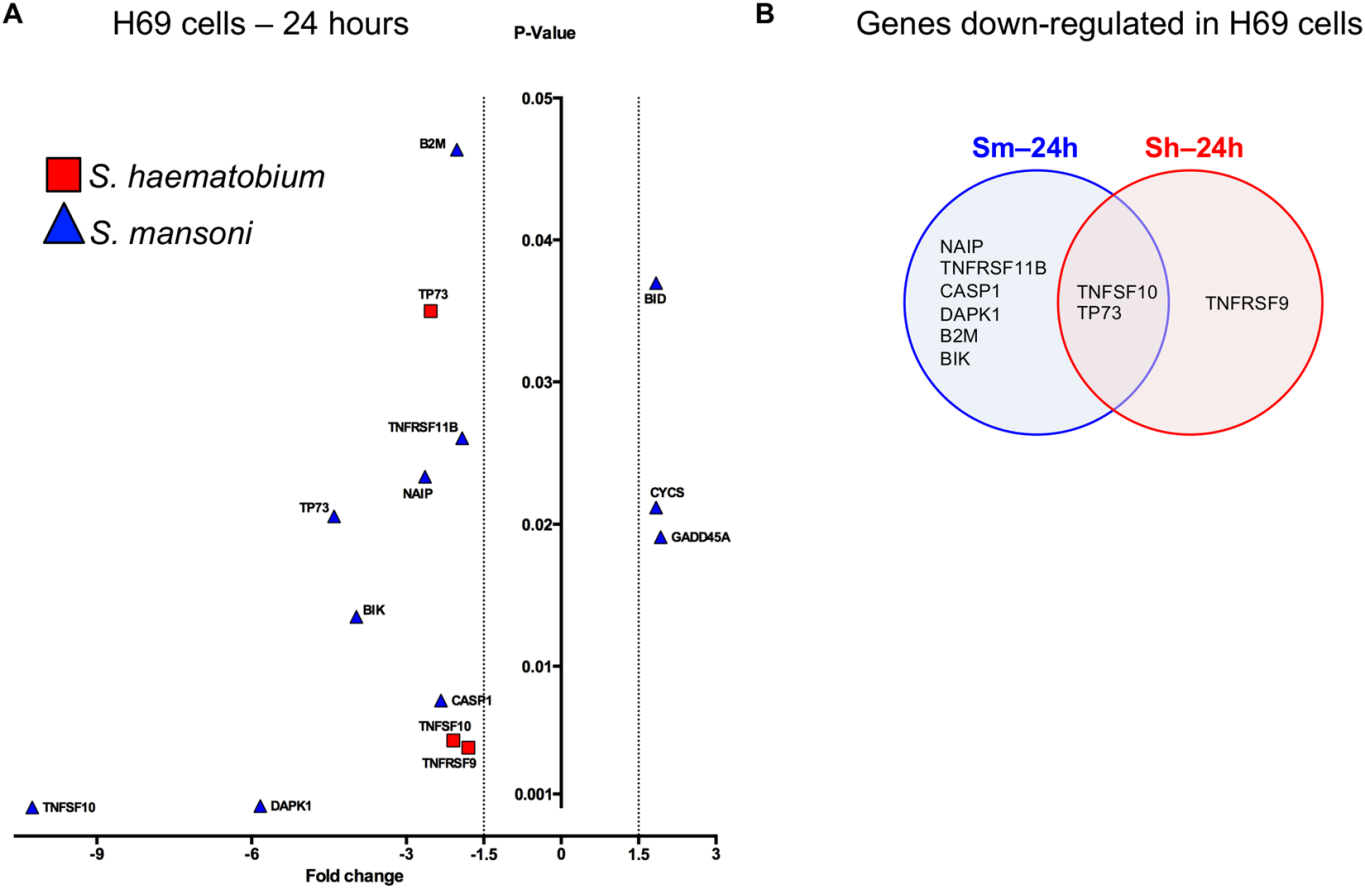
Schistosome eggs induced dysregulation of apoptosis-related genes in H69 cells. Volcano plot of the H69 cell gene response to non-contact co-culture with *S*. *mansoni* or *S*. *haematobium* eggs as indicated for 24 hours. Gene expression was measured using qPCR gene arrays designed to assess apoptosis-associated transcripts. Significantly dysregulated (*P* < 0.05) genes with >±1.5-fold-change, are shown. (Table S1 includes the complete data set of gene expression changes.) Panel B. Venn diagram compiled only with the H69 cell culture conditions that share differentially expressed genes (DEG) – only downregulated genes were shared between the two conditions indicated.

Given schistosome eggs inhibited the cell growth of the H69 human cholangiocyte cell line, an effect that became evident after ~24 hours post-incubation (above), an apoptosis gene array was employed to evaluate the gene expression dysregulation in cells exposed to schistosome eggs for 24 hours. Twelve genes were dysregulated when the cells were co-cultured with *S*. *mansoni* and *S*. *haematobium* eggs (Fig. 3A). Whereas both TNFSF10 and TP73 genes were downregulated by both schistosome species (Fig. 3B), *S*. *mansoni* eggs induced upregulation of three genes, i.e. BID, CYCS and GADD45A, and *S*. *haematobium* eggs induced downregulation of TNFRSF9, TNFSF10 and TP73 (Table S3).

### Colorectal cancer signaling pathway was significantly dysregulated by *S*. *mansoni* but not *S*. *haematobium* eggs

**Figure 4:**
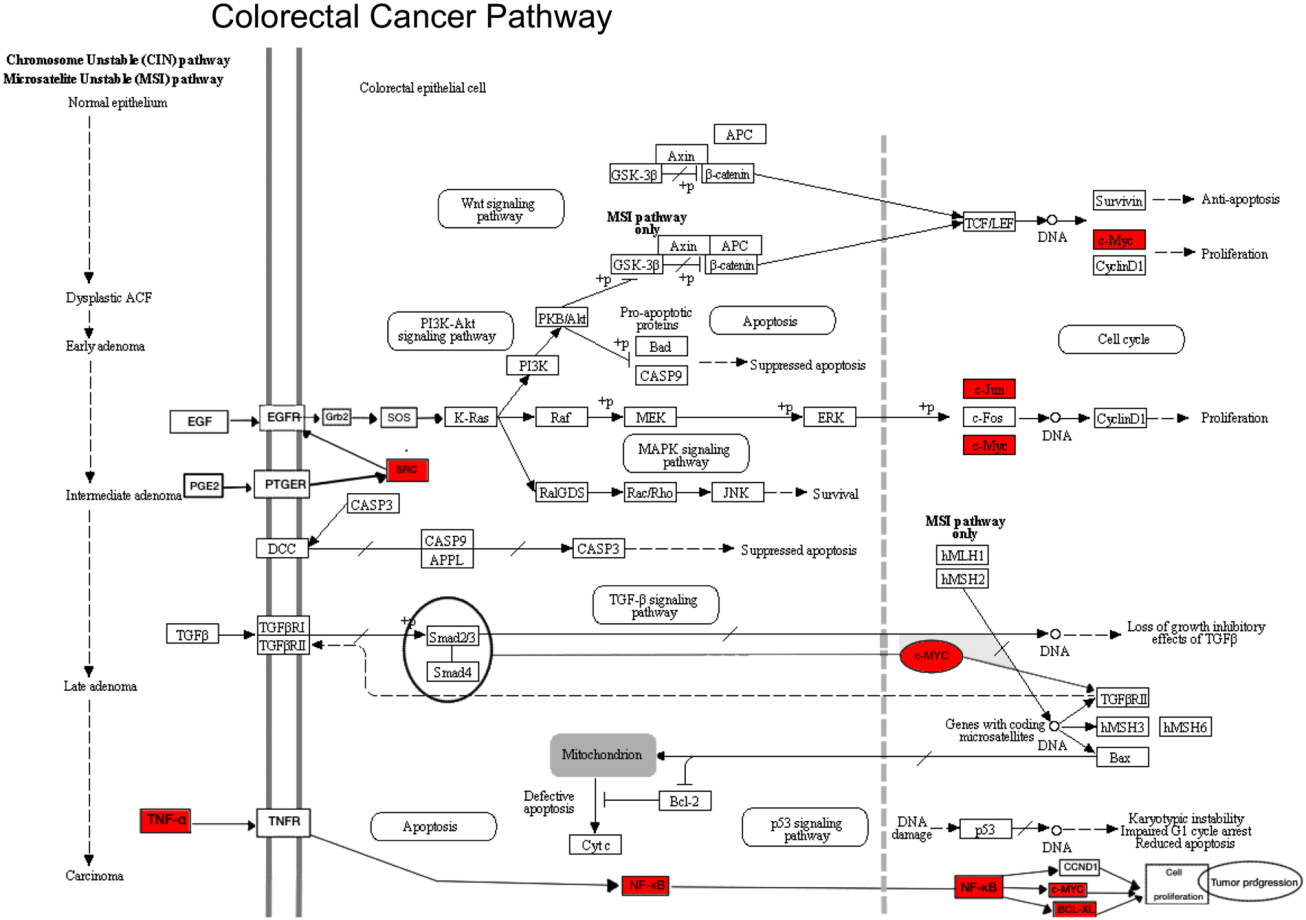
Significant dysregulation of genes involved in Colorectal Cancer Signaling Pathway in urothelial cells. The pathway was significantly dysregulated at early time point in *S*. *mansoni* eggs co-cultured HCV29 cells, and all the perturbed genes were upregulated (genes colored in red). Adapted from map05210 Colorectal cancer, KEEG Database^63–65^.

In order to detect significantly altered pathways in cells exposed to schistosome eggs a functional pathway analysis of differentially expressed genes was performed. The functional pathway analysis takes into consideration the differentially expressed genes detected in the gene expression arrays employed herein (see Methods) and determines which biological functions and canonical pathways are significantly associated with these dysregulated genes. At the early time point, i.e. 2 h after incubation of urothelial cells in the presence of *S*. *mansoni* eggs, genes associated to colorectal cancer metastasis signaling pathway were upregulated (Fig. 4)^19^. This pathway was significantly upregulated (with a positive z-score of 7.5 and *P* = 2.58 × 10^−08^) showing a cascade of significantly overexpressed genes (Table 1) including TNF, a pro-inflammatory cytokine implicated in colorectal cancers^20^ upregulated 9.72 fold, the oncogenes MYC and JUN upregulated more than 4 fold, among others (Table 1). Interestingly, urothelial cells co-cultured with *S*. *mansoni* eggs or *S*. *haematobium* eggs for 24 hours showed upregulation of WNT5B or both WNT5a and WNT5b, respectively (Fig. 2B,C). Notably, upregulation of WNT5a and WNT5b has been implicated in colon rectal cancer via non-canonical WNT signaling pathway^21^ However, the signaling pathway analysis did not support any significant correlation between the upregulated *wnt* genes in cells cultured with schistosome eggs and colorectal cancer pathway 24 hours after exposure.

**Table 1:** Differentially expressed genes associated with Colorectal Cancer Signaling Pathway identified in HCV29 cells exposed to eggs of *Schistosoma haematobium* (*Sh*) or *S*. *mansoni* (*Sm*).

Gene name (ID)	HCV29 cell line^*^		Gene description	Reference
	*Sh* eggs	*Sm* eggs		
TNF (Tumor Necrosis Factor)	NS	↑ 9.72 (*P* = 0.031) 2 h	Pro-inflammatory cytokine implicated in colorectal cancers, associated with tumor growth, proliferation and metastasis	^19, 20, 66^
RUNX1 (Runt Related Transcription Factor 1)	NS	↑ 4.59 (*P* = 0.042) 2 h and 24 h	Transcription factor with tumor suppressor functions associated with growth promotion of skin, oral, breast and ovarian tumor cells.	^67^
MYC (Proto-Oncogene)	NS	↑ 4.01 (*P* = 0.044) 2 h	Transcription factor involved in cell proliferation, inhibition of cell differentiation and apoptosis. Over expressed in over 50% of all cancers, is considered a metastasis marker.	^68, 69^
JUN (Proto-Oncogene, AP-1 Transcription Factor Subunit)	NS	↑ 3.34 (*P* = 0.029) 2 h	Transcription factor involves in cell cycle progression, cell survival, transformation and proliferation. Over expression of JUN has been related to p53 repression.	^70^
NFKB1 (Nuclear Factor Kappa B subunit 1)	NS	↑ 2.82 (*P* = 0.039) 2 h	Transcription factor responsible for the regulation of ~300 genes involved with immune, growth and inflammation processes. Over expressed in many cancer types including colorectal, breast, liver, prostate and kidney.	^71, 72^
SRC (Proto-Oncogene, Non-Receptor Tyrosine Kinase)	NS	↑ 2.72 (*P* = 0.006) 2 h	Proto-oncogene over expressed in colorectal, pancreas and prostate cancer, implicated in tumor progression, cell migration, angiogenesis, and metastasis.	^73, 74^
BCL2 (Apoptosis regulator)	NS	↑ 2.69 (*P* = 0.035) 2 h	Anti-apoptotic regulator involved in several lymphomas. When over expressed, it has been associated with low survival of diffuse large B-cell lymphoma	^75– 77^

^*^Fold change and *P* value, upregulation (↑), downregulation (↓), NS: non-significant. The exposure time of cells co-cultured with indicated schistosome eggs (2 h and/or 24 h) for which the specific gene expression dysregulation was detected is indicated after  the Fold change.

### Tumor suppressor P53-associated pathway was downregulated by both *S*. *mansoni* and *S*. *haematobium* eggs

**Figure 5:**
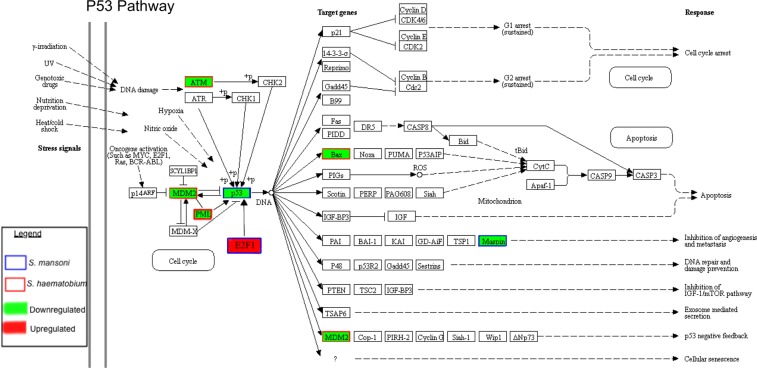
Significant dysregulation of P53 pathway in urothelial cells exposed to either *S*. *haematobium* or *S*. *mansoni* eggs for 24 hours. P53 pathway highlighting upregulated or downregulated genes in red or green, respectively. Genes affected by *S*. *mansoni* or *S*. *haematobium* are indicated by blue or red squares, respectively. Adapted from map04115 p53 signaling pathway, KEEG Database^63–65^.

The tumor suppressor P53-associated pathway was significantly downregulated in urothelial cells exposed to both *S*. *mansoni* and *S*. *haematobium* eggs. Notably, different sets of genes within the pathway were altered by the two schistosome species (Fig. 5 and Table 2), i.e. *S*. *mansoni* reduced the expression of P53 (fold change of −1.92, *P* = 0.0033), E2F1 (fold change of 2.75, *P* = 0.0268) and SERPINB5 (fold change of −3.09 and *P* = 0.0032) genes, whereas *S*. *haematobium* induced downregulation of ATM (fold change of −2.69, *P* = 0.0433), MDM2 (fold change of −2.64, *P* = 0.0187), PML (fold change of −2.50, *P* = 0.0231) and the BCL2-Associated X (BAX) (fold change of −2.22 and *P* = 0.0178) (Table 2). Two of these proteins were selected to validate the gene array findings at the protein level, i.e. ELISAs were performed to quantify P53 and BAX in urothelial cells co-cultured in the absence or presence of *S*. *mansoni* or *S*. *haematobium* eggs for 2 or 24 hours (Fig. S4). In concordance with the gene array findings, no significant changes were observed at 2 hours after egg-exposure for both BAX and P53 proteins among the groups of cells exposed to no eggs, *S*. *mansoni* or *S*. *haematobium* eggs (Fig. S4). As expected, at 24 hours post-exposure no significant differences were observed between *S*. *mansoni* egg-exposed cells (Sm 24 hr) and control (Control 24 hr) for BAX protein; however, a higher level of this protein was quantified in cells exposed to *S*. *haematobium* eggs (Sh 24 hr). Similar findings were obtained for P53 (Fig. S4).

**Table 2:** Differentially expressed genes associated with P53 Signaling Pathway identified in HCV29 cells exposed to either eggs of *Schistosoma haematobium* (*Sh*) or *S*. *mansoni* (*Sm*).

Gene name (ID)	HCV29 cell line*	Gene description		Reference
	*Sh* eggs	*Sm* eggs		
SERPINB5 (Serpin Family B, member 5)	NS	↓ −3.09 (*P* = 0.003) 24 h	Inhibitor of cancer cell invasion, metastasis and angiogenesis. Under expression associated with breast, prostate, thyroid and skin tumors.	^78^
ATM (Ataxia Telangiectasia Mutated, Serine/Threonine kinase)	↓ −2.69 (p = 0.043) 24 h	NS	Mediator in kinase cascade that controls DNA damage response, cell-cycle progression, DNA recombination and apoptosis. Its downregulation inhibits p53 and cell cycle arrest.	^79, 80^
MDM2 (MDM2 Proto-Oncogene)	↓ −2.64 (*P* = 0.018) 24 h	NS	Ubiquitin E3 ligase that degrades P53 is under the control of p53 in a regulatory feedback loop (Fig. 4)	^81, 82^
PML (Promyelocytic Leukemia)	↓ −2.50 (p = 0.023) 24 h	NS	Nuclear protein involved in cell cycle progression, DNA damage response, and apoptosis, i.e. key regulator in the p53 pathway. PML-deficient mice exhibit apoptotic defects.	^83– 85^
BAX (BCL2 Associated X, Apoptosis Regulator)	↓−2.22 (*P* = 0.017) 24 h	NS	Major promoter of apoptosis is regulated by p53, it has been involved in tumorigenesis by interfering with cell death.	^86^
P53 (Tumor Protein P53 or TP53 as in Fig. 2)	NS	↓ −1.92 (*P* = 0.003) 24 h	Tumor suppressor, a key transcription factor inhibiting cancer development, being inactivated in most tumors. It responds to cell stress by activating genes responsible for DNA repair, cell cycle arrest, anti-angiogenesis, apoptosis and autophagy	^87, 88^
E2F1 (E2F Transcription Factor 1)	NS	↑ 2.75 (*P* = 0.026) 24 h	Transcription factor that regulates cell cycle progression, involved in either oncogenesis or tumor suppression depending on cellular signals. E2F1 over expression associated with oncogenic transformation in rodent embryonic fibroblasts and tumorigenesis.	^89, 90^

**Fold change and *P* value, upregulation (↑), downregulation (↓), NS, non-significant. The exposure time of cells co-cultured with indicated schistosome eggs (2 h and/or 24 h) for which the specific gene expression dysregulation was detected is indicated after  the Fold change.

### Estrogen receptor and beta-estradiol altered by schistosome eggs

**Figure 6:**
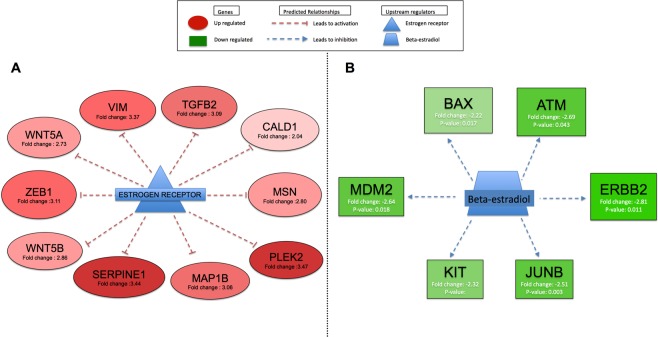
Upstream regulatory analysis (URA) of differentially expressed genes (DEGs) predicted the estrogen receptor and beta-estradiol (*P* < 0.05) to be inhibited in HCV29 cells co-culture for 24 h with *S*. *haematobium* eggs. Panel A. Significantly upregulated genes are shown in red. These genes, negatively regulated (T-symbol) by the estrogen receptor, were found to be upregulated in HCV29 cells co-cultured with *S*. *haematobium* eggs for 24 hours; red dashed lines indicate that these genes were activated. Therefore, the URA analysis significantly predicted the estrogen receptor as an inhibited upstream regulator of this set of genes in this dataset. Panel B. Significantly downregulated genes are shown in green. These genes, positively regulated (arrow) by beta-estradiol, were found to be downregulated in HCV29 cells co-cultured with *S*. *haematobium* eggs for 24 hours; blue dashed lines indicate that these genes were inhibited in our dataset. Therefore, the URA analysis significantly predicted beta-estradiol as an inhibited upstream regulator of this set of genes in the dataset.

Notably, the estrogen receptor (ESR1) was upregulated 2.68-fold (*P* = 0.000812) in urothelial cells co-cultured with *S*. *mansoni* eggs at the early time point (Fig. 2A), but not with *S*. *haematobium* eggs. However, the upstream regulatory analysis (URA) of differentially expressed genes predicted both the estrogen receptor and beta estradiol to be inhibited in urothelial cells co-cultured with *S*. *haematobium* eggs for 24 hours, but not with *S*. *mansoni* eggs (Fig. 6).

## Discussion

Infectious diseases are associated with >20% of cancers in the developing world^22^. About a dozen pathogens including human papillomavirus, Epstein-Barr virus and hepatitis B virus are well known agents of cancer-associated infections. Infections with several parasitic flatworms are also associated with carcinogenesis^5,22^. The International Agency for Research on Cancer (IARC) categorizes infection with the food-borne trematodes *Opisthorchis viverrini* and *Clonorchis sinensis*, both liver flukes, and the blood fluke *Schistosoma haematobium* as group 1 biological carcinogens^5^. More recently we have shown that the chronic infection with the liver fluke *Opisthorchis felineus* might also be associated with liver cancer^23^. In addition to parasitism directly damaging development, health and prosperity of infected populations, chronic infection with these liver and blood flukes leads to cholangiocarcinoma (CCA) (bile duct cancer) and squamous cell carcinoma (SCC) of the urinary bladder, respectively^5^. By contrast, chronic infection with phylogenetic relatives, including trematodes of the phylum Platyhelminthes which are major pathogens, has not been proven to be carcinogenic. These noteworthy differences suggest that either helminth-specific metabolites contribute to tumorigenesis and/or that certain tissues or organs directly or indirectly exposed to the parasite and/or parasite-derived metabolites are particularly susceptible to infection-induced malignancy^24^. Consequently, aiming to compare/contrast the effect of eggs of a well-established carcinogenic species of schistosome, i.e. *S*. *haematobium*, with eggs of a non-carcinogenic schistosome, i.e. *S*. *mansoni*, established epithelial cells derived from two discrete tissues, HCV29, from human urothelium, and the H69, from cholangiocytes, were cultured in the presence of eggs of *S*. *haematobium* or *S*. *mansoni*. Remarkably, epithelial cells from urinary and biliary tract displayed differential responses to *S*. *haematobium* and *S*. *mansoni*. The proliferative response and associated changes in signaling pathways responses of these cells appear to have been influenced by both the tissue origin of the epithelial cells and the schistosome species.

Both *S*. *haematobium* and *S*. *mansoni* eggs stimulated cellular growth in HCV29 urothelial cells when compared to control cells in the absence of schistosome eggs. Moreover, genes strongly associated with cell migration (PTK2 and VIM)^25^, proliferation (AKT1)^26^, inhibition of apoptosis (SNAI2)^27^, and carcinogenesis mediated by the TGFβ signaling pathway (SMAD2 and ZEB2)^28^ were upregulated in cells exposed to both species. Therefore, it is tempting to speculate that the carcinogenicity of the chronic infection with these parasites not only depends on the schistosome species but also the host tissue exposed to antigens and excretory-secretory products from the eggs. More studies, including *in vitro* cell co-culture experiments with informative cell lines derived from different tissues will contribute to the resolution of this issue. Several cancer-inducing parasites stimulate cellular proliferation when co-cultured with mammalian cells. Excretory-secretory products (ES) from the liver flukes, *O*. *viverrini* and *C*. *sinensis* promoted growth of mouse fibroblast NIH-3T3 and human kidney embryonic epithelial cells HEK293^29,30^. *S*. *haematobium* egg antigens induced cell proliferation, apoptosis inhibition and altered cell migration *in vitro*^31^. In addition, cells pretreated with *S*. *haematobium* egg antigens induced tumor development when injected in immunodeficient mice^32^. Moreover, intravesical administration of *S*. *haematobium* total antigen into CD-1 mice induces a high incidence of urothelial dysplasia^33^. Other reports reveal that human urothelial and bovine endothelial cells exhibit increased proliferation following exposure to soluble egg antigen of *S*. *haematobium*^34^. *S*. *mansoni* also promotes proliferation of some cell lineages as granulomas are a rich source of cytokines and growth factors and stimulates tissue proliferation and fibroplasia in mammalian tissue^35^ and total antigen from *S*. *mansoni* egg stimulates fibroblasts *in vitro*^36^. *S*. *mansoni* soluble egg antigens induce proliferation of H69 cells and murine cholangiocytes^37^. However, herein we showed that when each of the species of schistosome eggs were co-cultured with the H69 cholangiocyte cell line a consistent decrease in cellular proliferation, even using heat-inactivated schistosome eggs, was observed.

Schistosome soluble egg antigens stimulate cellular proliferation of murine splenocytes whereas higher concentrations induce apoptosis in a dose-dependent fashion^38^. Antigens from both *S*. *japonicum* and *S*. *mansoni* negatively regulate hepatic stellate and CD4 + ve T cells^39,40^. Some of the disparities among these reports may relate to methods to prepare egg-secreted proteins (ESP) or soluble egg antigens (SEA). Egg-secreted proteins differ significantly when compared to soluble egg antigen in terms of protein sizes and the presence of proteases that are found in ESP but not in SEA^41^. In addition, we cannot completely rule out the effect observed in the current study was due to a foreign body co-cultured with the cells. However, we speculate that if that was the case the same unspecific effect should have been observed on both cell lines regardless the schistosome species. In this regard, the findings showed (1) species-specific effects on the same cell line, e.g. even though both schistosome eggs stimulated cell proliferation of HCV29, *S*. *haematobium* eggs induced more proliferation than *S*. *mansoni* eggs, and (2) differences on effect intensities between live and heat-killed eggs, e.g. even though similar effects were induced by heat-killed or live *S*. *mansoni* eggs, the latter clearly had a stronger effect on both stimulating HCV29 cells and inhibiting H69 cells. By contrast, *S*. *haematobium* heat-killed eggs slightly induced a stronger effect on the cells compared to live eggs. These differences induced by the two schistosomes on different cells presumably would not have been detected if the effects were due to just the presence of a foreign inert body. Last, there was increased proliferation when the cells were cultured in the presence of ES only collected from the eggs, i.e. no eggs were used in the experiment. This strongly suggested the effect was not due to the presence of an inert body in the experimental setting.

The Ingenuity Pathway Analysis (IPA) identified the colorectal metastasis-signaling pathway significantly dysregulated in urothelial HCV29 cells co-cultured with *S*. *mansoni* eggs, but not with *S*. *haematobium* eggs after being co-cultured for 2 hours. In contrast, exposure of the urothelial cells to *S*. *haematobium* eggs for 24 hours induced upregulation of WNT5a and WNT5b. Although not significantly associated to Colorectal cancer pathway in our analysis, these genes have been involved in carcinogenesis, including colorectal cancer *via* non-canonical WNT signaling pathway^21^. On the other hand, *S*. *mansoni* eggs induced upregulation of these same genes (WNT5a and WNT5b) at both time points. A link has been proposed between infection with *S*. *mansoni* and colorectal cancers^42^. IARC classifies infection with *S*. *haematobium* as a group 1 carcinogen, whereas infection with *S*. *mansoni* is included in group 3, indicating there is insufficient evidence to determine its carcinogenicity^5^. Nonetheless, schistosomal colitis is commonly associated with earlier onset of multicentric colorectal cancer, and notably mucinous adenocarcinoma often presents at an advanced stage in regions endemic for infection with *S*. *mansoni*^43^. Moreover, a higher incidence of altered p53 expression with schistosomal colitis-associated colorectal cancer suggests an association between schistosomiasis and alterations in *p53* activation^43^. Intestinal schistosomiasis caused by *S*. *japonicum* exhibits a geographic distribution that overlaps with populations affected by early onset of colon cancer in China^44^. In addition, several genes were upregulated in HCV29 cells at the early time point when exposed to *S*. *mansoni* eggs. Interestingly, only *β-2 microglobulin* (B2M) was downregulated 1.5 folds and more than 3 folds 2- and 24-hours post-exposure, respectively. B2M belongs to the major histocompatibility complex (MHC) class I^45^. Because B2M is not only markedly downregulated in colorectal cancer tissue compared with normal mucosa^46^, but also plays a prominent role in antigen presentation, it is tempting to speculate that its downregulation might impede host clearance of the fluke infection.

Surprisingly, significantly dysregulated pathways were not identified in HCV29 cells co-cultured with *S*. *haematobium* eggs at the early time point (2 hours); FOXD3 was the only gene to be upregulated. This transcription factor is a member of the forkhead family with a key role in progenitor cell pluripotency and differentiation, and regulation of cellular transition from naïve to primed pluripotency^47^. FOXD3 is upregulated in renal cancer and endometrial tumors and downregulated in cancers of the colon and cervix^48^. Remarkably, by 24 hours post-exposure not only were  many more genes were upregulated in HCV29 cells exposed to *S*. *haematobium* eggs, but also the number of dysregulated genes outnumbered those dysregulated by *S*. *mansoni* eggs. It is tempting to speculate that this is related to the oncogenic nature of *S*. *haematobium* infection, although further studies are needed to clarify this point. Several genes dysregulated by either or both species of schistosomes have been linked to inflammation and schistosomiasis-associated disease but have not been associated with cancer, at least in a direct manner, e.g. Tumor Necrosis Factor (TNF) or Nuclear Factor Kappa B subunit 1 (NFK-B). Notably, elevated TNF levels can occur during acute schistosomiasis and severe pathology is associated with elevated circulating levels of TNF and TNF receptors (TNFRs). Moreover, TNF might be involved in maintaining *S*. *mansoni* adult viability in the portal system^49^, and would be required by the parasite for egg-laying and egg-excretion from the host^50^. Consistent with this hypothesis, here TNF was upregulated >9 folds by eggs of *S*. *mansoni* but not *S*. *haematobium*.

Estradiol-like metabolites have been characterized both in *S*. *haematobium* parasites and in urine during urogenital schistosomiasis^9^. We have recently predicted pathways and enzymes that are involved in the production of these metabolites and emphasized their potential effects on the dysregulation of the tumor suppressor gene p53 expression during urogenital schistosomiasis (reviewed in^51^). Mutations of p53 in the context of UGS-induced bladder cancers from Angola along with sialylated glycans have been suggested as surrogate biomarkers of bladder carcinogenesis associated with *S*. *haematobium* infection highlighting links between infection and cancer development^52^. Eggs of *S*. *haematobium* express the sialyl-Lewis sLe^a^ and sLe^x^ antigens in mimicry of human leukocytes glycosylation, which may play a role in the colonization and disease dissemination. Tumor suppressors including P53 and genes involved in the P53 pathway and metalloprotease inhibitors were downregulated when exposed to schistosome eggs. In bladder cancers, recurrent mutations occur in >30 other genes involved in cell proliferation, differentiation, genetic stability, and specifically cell-cycle regulation, chromatin regulation, and kinase signaling pathways^53^. The downregulation of *p53* is characteristic of cancers in general and SCC of the bladder in particular. In this regard, we found the tumor suppressor P53-associated pathway significantly downregulated in urothelial cells exposed to both *S*. *mansoni* and *S*. *haematobium* eggs. The P53 signaling pathway is responsible for hundreds of downstream transcriptional targets involved in the inhibition of cell growth, induction of apoptosis and regulation of metabolism in response to oncogene activation and other signals^54^. In a mouse model of infection-related bladder cancer, *S*. *haematobium* egg-induced bladder urothelial abnormalities were dependent on p53 in a host sex dependent manner^10,14^. We decided to validate the dysregulation of P53 and BAX proteins performing ELISA assays, key genes detected in our dataset involved in carcinogenesis. The protein quantification for BAX and P53 concurred with the gene array data 2 hours after exposure. In addition, as in the gene array data BAX protein was not dysregulated in *S*. *mansoni* egg-exposed cells at 24 hours post-exposure. However, BAX and P53 proteins were over expressed in *S*. *haematobium* egg-exposed cells and P53 was not down-regulated in *S*. *mansoni* egg-exposed cells. Among confounders that might explain the unexpected differences, different batches of eggs and cells employed in the two experiments, which were separated widely in time. In addition, recent studies highlight the lack of correlation between mRNA and protein levels for many genes, including those involved in carcinogenesis. This would reflect the complexity of the gene expression regulation  at several levels including post-transcriptional regulation of mRNA and post-translational modifications of proteins^55,56^. The latter could also explain the inconsistencies between the qPCR gene array signals for the bax and p53 genes and concentrations of BAX and P53 in cells exposed to schistosome eggs. Future studies involving high throughput transcriptomic and proteomic analyses from cells exposed to eggs and excretory-secretory products from eggs may clarify these contradictory findings.

Upstream regulatory analysis (URA) of differentially expressed genes (DEGs) predicted that the exposure of urothelial cells to eggs of *S*. *haematobium* but not *S*. *mansoni* downregulated expression of the estrogen receptor. This phenomenon was previously reported in other experimental models showing that an estradiol-like molecule antagonist to estradiol represses the transcriptional activity of the estrogen receptor (ER)^57^. We have reviewed how an estrogen-DNA adduct mediated pathway may be involved in the pathogenesis of the squamous cell carcinoma of the bladder associated with urogenital schistosomiasis^58,59^. Extracts of *S*. *haematobium* eggs induced tumor-like phenotypes in cultured cells and, in addition, estrogen-derived, reactive metabolites occurred in this pathogen and in sera during UGS including catechol estrogen quinones (CEQ) and CEQ-DNA-adducts^9^.

To conclude, H69 and HCV29 cells displayed differential responses to eggs of *S*. *haematobium* and *S*. *mansoni*, supporting the hypothesis that the carcinogenic nature of infection with *S*. *haematobium* is related to its interaction with the specialized epithelium of the human bladder. Proliferative responses of these cells were influenced by both the tissue origin of the epithelial cells and the schistosome species. Focused studies of the molecular mechanisms underlying the association between UGS and SSC of the bladder are expected to enhance prospects to manage this neglected tropical disease-related cancer.

## Materials and Methods

### Ethics statement

Male LVG hamsters were purchased from Charles River (Wilmington, MA) and maintained at the Biomedical Research Institute’s (BRI), Rockville, MD, animal facility accredited by the American Association for Accreditation of Laboratory Animal Care (AAALAC; #000779). BRI is a USDA registered animal facility (51-R-0050), and has an Animal Welfare Assurance on file with the National Institutes of Health, Office of Laboratory Animal Welfare (OLAW), A3080-01. Maintenance of hamsters exposed to Egyptian strain of *S*. *haematobium* cercariae and harvesting of tissues were approved by the BRI Institutional Animal Care and Use Committee. All procedures employed were consistent with the Guide for the Care and Use of Laboratory Animals.

Mice infected with the NMRI (Puerto Rican) strain of *Schistosoma mansoni* were supplied by BRI and maintained at The George Washington University (GW) following protocols approved by the GW Institutional Animal Case and Use Committee.

### Schistosome eggs and excretory-secretory products (ES) from eggs

Isolation of eggs from liver of experimentally-infected rodents was performed as previously described^60^. In brief, 3–5 dissected livers from *S*. *mansoni-*infected mice, and 2–3 livers from *S*. *haematobium-*infected Syrian golden hamsters were briefly washed in 70% ethanol solution, chopped finely with a sterile scalpel blade and transferred to a 50 ml conical tube containing 45 ml phosphate-buffered saline (1X PBS), 2% antibiotic-antimycotic solution (Life Technologies) and 0.5% clostridial collagenase (Sigma). The minced livers were incubated with gentle shaking overnight at 37 °C, centrifuged at 400 g for 5 min, and washed with 1X PBS 3 times. After the last wash, the pellet was resuspended in 25 ml 1X PBS, passed successively through sterile 250 μm and 150 μm sieves, and the filtrate centrifuged at 400 g for 5 min. The liver mixture filtrate mixture was centrifuged at 400 g for 5 min, the supernatant discarded and the pellet resuspended in 3 ml of 1X PBS, applied to a column of Percoll, prepared by mixing 8 ml of Percoll (GE Healthcare Bio-Science AB) with 32 ml of 0.25 M sucrose, and centrifuged at 800 g for 10 min. Liver cells and debris that remained on the top of the Percoll were removed with a Pasteur pipette. The schistosome eggs, which pelleted tightly at the bottom of the tube, were washed three times with 1X PBS to remove residual host tissues. Further purification was achieved by resuspending the eggs in 0.5 ml of 1X PBS and a second Percoll column, prepared by mixing 2.5 ml of Percoll with 7.5 ml of 0.25 M sucrose. The eggs were pelleted, washed as before, counted and cultured in DMEM, 10% FBS and 2% antibiotic-antimycotic at 37 °C, 5% CO_2_. Egg viability was evaluated by hatching an aliquot of a few hundred eggs in water under light. When heat-killed eggs were included as controls, eggs were transferred to a 1.5 ml tube and incubated for 10 min at 80 °C in a heat-block. For excretory-secretory products (ES), eggs were washed 3 times in 1X PBS, resuspended in DMEM, 2% antibiotic-antimycotic solution and protease inhibitor cocktail (Amresco), and incubated in 2 ml of serum-free media in 12-well plates overnight at 37 °C, 5% CO_2_. The supernatant (i.e., ES) was collected, clarified by centrifugation at ~14,000 rpm, 4 °C, 30 min, transferred to a new tube where additional protease inhibitor cocktail was added and stored at −80 °C. Protein concentrations of ES was determined using the bicinchoninic acid assay (BCA kit, Pierce, Rockford, IL).

### Human epithelial cell lines

The human cholangiocyte H69 cell line is a SV40-transformed human bile duct epithelial cell line derived from a normal liver^61^. The H69 cells were used between passages 10 and 30 and grown in H69 medium that included 43.82% DMEM medium (Dulbecco’s Modified Eagle’s Medium), 43.82% DMEM/Ham’s F12, 10% fetal bovine serum (FBS), supplemented with 1% adenine, 0.1% insulin, 0.1% epinephrine, 0.1% T3-T, 0.03% epidermal growth factor (EGF), 0.03% of a 4% hydrocortisone dilution in DMEM and 1% penicillin/streptomycin (PS). The human HCV29 cell line is derived from non-malignant urothelium of the ureter^62^. The HCV29 cells, used between passages of 10 to 30, were maintained in DMEM supplemented with 10% FBS and 1% PS (complete DMEM). Passages of both cell lines were undertaken when the confluence reached 90% in a 75 cm^2^ flask in a 1/10 dilution using trypsin to dislocate cells. Both cell lines tested negative for mycoplasma (not shown).

### Real time assessment of cell proliferation by xCELLigence system

Cellular proliferation of H69 and HCV29 cells was assessed using the xCELLigence DP system (ACEA Biosciences, San Diego, CA, USA), designed to monitor events in real time by measuring electrical impedance across inter-digitated microelectrodes integrated on the bottom of tissue culture E-plates, see http://www.aceabio.com/main.aspx^16^. H69 cells were seeded at 5,000 cells/well in E-plates in H69 medium, cultured for one day, rinsed in 1X PBS and cultured in 1/20 diluted H69 medium in 50% DMEM and 50% DMEM/Ham’s F12, containing 1% of PS, i.e. 0.5% FBS final concentration, as described^17^. For HCV29 cells a cell number titration experiment was first performed (Fig. S1), being 20,000 cells/well the selected number of cells for subsequent experiments. HCV29 cells were seeded in E-plates in complete DMEM medium, cultured for one day, rinsed with 1X PBS, and cultured in 1/20 diluted DMEM media. Both cell lines were fasted in 1/20 diluted medium for 4–6 h after which the cells were co-cultured with viable or heat-killed (below) schistosome eggs. The eggs were placed in a tissue culture insert transwell where the base of the insert is a polyethylene terephthalate (PET) membrane with a median pore size of 0.4 μm (ACEA catalog no. 06465382001). The transwell carrying the eggs were inserted into tissue culture plates were the cells were growing. Consequently, cells cultured on plastic in the tissue culture wells were exposed to the excretory-secretory products (ES) diffusing from the insert chamber carrying the eggs. For experiments where HCV29 cells were cultured in the presence of ES previously collected from *S*. *mansoni* or *S*. *haematobium* eggs as described above, the cells were fasted in 100 μl of 1/20 diluted medium for 4–6 hours in the xCELLigence platform after which 100 μl of 2X ES of the desire final concentration, i.e. no ES (control), 10, 2 or 0.4 μg/ml, was added to the well. The cellular growth was monitored in real time for at least 60 hours. The normalized cell index (CI), obtained by dividing the CI value at each time point by the CI at the time of addition of the eggs was ascertained with the assistance of the RTCA Software 1.2 (ACEA). Normalized CI values were imported into Microsoft Excel for analysis where the cellular growth of cells exposed to the eggs was expressed as the percentage of the normalized CI of cells cultured with indicated eggs compared to (i.e. divided by) that of control cells cultured in 1/20 diluted media. (Control cells growth rate = 100%.) Statistical significance among the groups was assessed using Analysis of Variance (ANOVA) and Student’s *t*-test. *P* values of ≤0.05 were considered significant.

### Epithelial cells co-cultured with schistosome eggs

H69 and HCV29 cells were seeded into wells of 6-well plates at a cell density ranging from 2.5 × 10^5^ and 3 × 10^5^ cells/well in 2 ml media and cultured until 80–90% confluence (usually 12–24 h) before being co-cultured with schistosome eggs. Before the addition of the eggs, the cells were starved for at least four hours in 1/20 dilution with 1% PS of their respective media. Thereafter, wells containing starved cells were fitted with Corning 6 Well Transwell Inserts, 0.4 μm PET Membrane, TC Treated, and ~2.5 × 10^3^ eggs of *S*. *mansoni* or *S*. *haematobium* were added to the Transwell. The Transwell ensured exposure of the cells to the excretory-secretory products (ES) of the eggs. Transwells were removed after two hours (‘early time point’) or 24 hours (‘late time point’) at which time cells were harvested for RNA isolation and gene expression analysis, or protein quantification. Cells not exposed to eggs but otherwise cultured similarly served as the control.

### ELISA-based quantification of selected human proteins

Human urothelial HCV29 cells exposed to schistosome eggs or control cells exposed to no eggs for 2 or 24 hours in 6 -well plates in triplicates were harvested for protein isolation and quantification of selected human proteins using commercially available ELISA kits (Abcam). Based on the qPCR gene array findings three human proteins were measured by ELISA, i.e. BAX (https://www.abcam.com/human-bax-elisa-kit-ab199080.html), MASPIN (SERPIN B5) (https://www.abcam.com/human-maspin-elisa-kitab218269.html), and P53 (https://www.abcam.com/human-p53-elisa-kit-ab171571.html). At the indicated time points the cell supernatant was removed and the cells washed twice with 1x PBS. The cells were exposed to 20μl of protease inhibitor cocktail (Calbiochem 539137), collected using a cell scraper and transferred to a 1.5 ml tube. One ml of 1xPBS was used to rinse the well and transferred to the corresponding tube that was kept on ice all the time. Thereafter, the cells were pelleted at 400 × g, for 10 min at 4 °C, and the cell pellet resuspended in 1 ml of 1xPBS. The cells were centrifugated as above and the pellet resuspended in 60μl of CEB PTR buffer (ELISA Abcam kit). The protein concentration for each sample was determined using the bicinchoninic acid assay (BCA kit, Pierce, Rockford, IL), and the input total protein concentration was 20 ng/ml for each reaction. The ELISA assays were performed following the manufacturer’s instructions. In brief, a colorimetric reaction became apparent after the addition of the TMB substrate to the protein lysate mixed with the protein-specific antibodies in a ‘sandwich’ format in a 96-well plate. Standard curves for each target protein were included in the assay as positive control and as reference for the quantification. Optical densities at 450 nm were determined using a spectrophotometer plate reader, and the raw readouts were exported and analysed using Graphpad Prism software. Three biological replicates with three technical replicates per biological repeat were carried out. Levels of MASPIN were below the level of detection of the kit.

### RNA isolation and gene arrays analysis

Total RNA was isolated from cells using the miRNeasy Mini kit (Qiagen) following the manufacturer’s instruction. Briefly, 700 μl of QIAzol Lysis Reagent was added to each cell pellet and homogenized using a sterile micropestle (Kontes, Vineland NJ). Chloroform, 140 μl, was added, contents mixed, and clarified at 12,000 × g, 15 min at 4 °C. The upper aqueous phase was transferred to a new tube containing 100% ethanol; subsequent washes through a spin column and on-column DNase treatment were performed. RNA was eluted in ~35 μl of RNAse-free water, and concentration, purity and integrity evaluated using spectrophotometry (Nanodrop 1000) and Agilent 2100 Bioanalyzer. The RNA preps were stored at −80 °C until processed for cDNA synthesis using 300 to 500 ng of total RNA and qPCR following the PrimerPCR Assay protocol (Bio-Rad). The qPCR experiments were performed using a Bio-Rad iCycler iQ5 with an initial activation step of 95 °C for 10 min followed by 40 cycles of 95 °C for 10 sec and 60 °C for 1 min. A melting curve analysis from 55 °C to 95 °C and 0.5 °C temperature increment every 30 sec was included at the end of the run. Specific gene arrays were selected; (1) Oncogenes and tumor suppressor genes (SAB Target List), http://www.bio-rad.com/en-uk/prime-pcr-assays/predesigned-plate/sybr-green-oncogenes-tumor-suppressor-genes-sab-target-list-h96; (2) Epithelial to mesenchymal transition (EMT) (SAB Target List), http://www.bio-rad.com/en-uk/prime-pcr-assays/predesigned-plate/sybr-green-epithelial-mesenchymal-transition-emt-sab-target-list-h96; and (3) Apoptosis (SAB Target List), http://www.bio-rad.com/en-uk/prime-pcr-assays/predesigned-plate/sybr-green-apoptosis-sab-target-list-h96. These predesigned arrays allow the detection of 84 genes along with controls that include DNA contamination control, positive PCR control, RNA quality control, and reverse transcription control. Ct values were exported and analyzed using Prime PCR Analysis software (http://www.bio-rad.com/en-us/sku/genestudy-1-0-030-1023-primepcr-analysis-software), and relative quantitation performed using the 2^−ΔΔCt^ method employing a panel of three housekeeping genes: glyceraldehyde-3-phosphate dehydrogenase, hypoxanthine phosphoribosyltransferase 1, and TATA-box binding protein. Control groups (cells exposed to media alone) were used as calibrator samples. Three biological replicates were performed. Venn diagrams were performed using the online free tool http://bioinformatics.psb.ugent.be/webtools/Venn/.

### Gene pathway analysis

Functional pathway and upstream regulatory analysis (URA) of differentially expressed genes (DEGs) was performed with the assistance of Ingenuity Pathway Analysis (IPA QIAGEN Redwood City, www.qiagen.com/ingenuity) software. In brief, the dataset from the real time PCR analysis was imported into the IPA to define the genes which were significantly up and downregulated and P-values were set up equal or lower than 0.05 as the significant threshold. IPA includes Benjamini-Hochberg analysis of multiple testing correction based on the Fisher’s exact test p-value to calculate false discovery rate between multiple comparisons. Additionally, IPA uses Right-Tailed Fisher’s Exact Test to determine which biological functions and canonical pathways are significantly associated with the genes of interest compared with the whole Ingenuity knowledge base (http://www.ingenuity.com/science/knowledge-base). In addition, to determine predictions about upstream processes, IPA provides a z-score, where z-scores >2 or <−2 are considered significant. This analysis considers the directional effect of one molecule or process on another, and the direction of this change in the dataset, i.e. it represents the statistical measure of the correlation between the direction of the change (up- or down- regulation) and gene expression (https://chhe.research.ncsu.edu/wordpress/wp-content/uploads/2015/10/IPA-Data-Analysis-training-slides-2016_04.pdf). The IPA analysis also enables the identification of biological networks, global functions and functional pathways for a particular dataset. The analysis estimates the significance of the genes in the network, the other genes with which it interacts, and how the products of the genes directly or indirectly act on each other, including those not involved in the analysis (determining up stream regulators). The networks are ranked depending on their number of significantly differentially expressed genes and associated relevant diseases. Genes in the network are represented as nodes, and the biological relationship between two nodes is represented as an edge (line). The intensity of the node color is related to the fold change of the differentially expressed genes, i.e. upregulated or downregulated genes indicated in red or green, respectively. All edges are supported by at least one published report and/or canonical definition in the Ingenuity Pathways Knowledge Base.

### Statistical analysis

For gene expression studies, fold change values of significantly DEGs (*P* ≤ 0.05) from the four analyzed gene arrays (above) using Prime PCR Analysis were exported to GraphPad Prism 6.02, pooled and plotted in a volcano plot. The genes with *P*-value ≤ 0.05 and a significant fold-change >1.5 or <−1.5 for upregulated and downregulated genes, respectively, were included in subsequent analyses.

## Supplementary information

Supplementary Information

Supplementary Tables
