# Supplementary material for: Differential responses of epithelial cells from urinary and biliary tract to eggs of Schistosoma haematobium and S. mansoni
Source: Sci Rep. 2019 Jul 24;9:10731. doi: 10.1038/s41598-019-46917-y (PMC6656753; doi:10.1038/s41598-019-46917-y)
Supplement: Supplementary file 1 — Supplementary Information [file 41598_2019_46917_MOESM1_ESM.pdf]

Supplementary information

Differential responses of epithelial cells from urinary and biliary tract to eggs of *Schistosoma haematobium* and *S. mansoni*

Rafael Nacif-Pimenta[1,2], Alessandra da Silva Orfanó[3], Ilana A. Mosley[1], Shannon E. Karinshak[1], Kenji Ishida[4], Victoria H. Mann[1], Paulo Marcos Zech Coelho[2], José M. Correia da Costa[5,6], Michael H. Hsieh[4,7,8], Paul J. Brindley[1]*, Gabriel Rinaldi[1]*

1. Department of Microbiology, Immunology & Tropical Medicine, and Research Center for the Neglected Diseases of Poverty, School of Medicine and Health Sciences, George Washington University, Washington, USA
2. Laboratório de Esquistossomose, Centro de Pesquisas René Rachou, Fundação Oswaldo Cruz - FIOCRUZ, Belo Horizonte, Minas Gerais, Brazil
3. Laboratório de Entomologia Médica, Centro de Pesquisas René Rachou, Fundação Oswaldo Cruz - FIOCRUZ, Belo Horizonte, Minas Gerais, Brazil
4. Biomedical Research Institute, Rockville, Maryland, USA
5. Department of Infectious Diseases, R&D Unit, INSA-National Health Institute Dr. Ricardo Jorge, Porto, Portugal
6. Center for the Study of Animal Science, ICETA, University of Porto, Portugal
7. Department of Urology, School of Medicine and Health Sciences, George Washington University, Washington, USA
8. Children's National Health System, Washington, District of Columbia, USA

***Correspondence.** Paul J. Brindley, email: pbrindley@gwu.edu and Gabriel Rinaldi, current address: Wellcome Sanger Institute, Wellcome Genome Campus, Hinxton, Cambridge, CB10 1SA, UK; Phone +44 (0) 1223 494864, email; gr10@sanger.ac.uk

Supplementary information

Figure S1. Cell titration assay for human urothelial cells HCV29. In order to analyze the real time cell proliferation kinetics for HCV29 cells, a cell titration assay seeding 2,500, 5,000, 10,000 and 20,000 cells per well was performed. Unprocessed data showing the cell index over time for each tested cell number per well as indicated are presented as displayed by RTCA Software 1.2 (ACEA). All curves represent the averages of three technical replicates for the experiment and standard deviations bars are shown at each data point.

Figure S2. Cell growth measured in real time using the xCELLigence platform of cells co-cultured with schistosome eggs. Unprocessed data of representative experiments showing the normalized cell index (Normalized CI) over time for HCV29 (Panel A) or H69 (B) cells co-cultured with *S. mansoni* eggs, *S. haematobium* eggs, or control cells co-cultured without eggs as indicated, are presented as displayed by RTCA Software 1.2 (ACEA). The normalization time is shown as a vertical black line, and the arrow indicated when eggs were added to the culture. All curves represent the averages of three technical replicates for the experiment and bar indicate the standard deviation at each data point.

Figure S3. Cell growth measured in real time using the xCELLigence platform of cells co-cultured with schistosome egg excretory-secretory products (ES). HCV29 cells cultured in the presence of indicated concentrations of *S. mansoni* egg ES (left panel) or *S. haematobium* egg ES (right panel) over time after the addition of ES. Cell growth was expressed as percentage of the Normalized Cell Index of cells cultured with ES compared with control cells cultured in 1/20 diluted media without ES (control cell growth rate = 100%). All curves represent the averages of two technical replicates for the experiment and standard deviation bars are shown at each data point. *Sm* egg ES: *S. mansoni* egg excretory-secretory products, *Sh* egg ES: *S. haematobium* egg excretory-secretory products.

Figure S4. Changes in the concentration of selected human proteins induced by co-culturing schistosome eggs and human urothelial cells for 2 or 24 hours. Concentrations of the human BAX (panel A) or P53 (panel B) proteins measured by ELISA assays in the indicated experimental conditions. *Sh*: *Schistosoma haematobium* eggs, *Sm*: *Schistosoma mansoni* eggs. * indicates P≦0.05, ** indicates P≦0.01, NS indicates non-significant.

Table S1. Significant differentially expressed genes (DEG) in all tested conditions, indicating upregulated and downregulated genes, fold change and P value. NS: non-significant

Table S2. List of all the genes indicated in the Venn diagram shown in Figure 2C.

Supplementary Figure S1

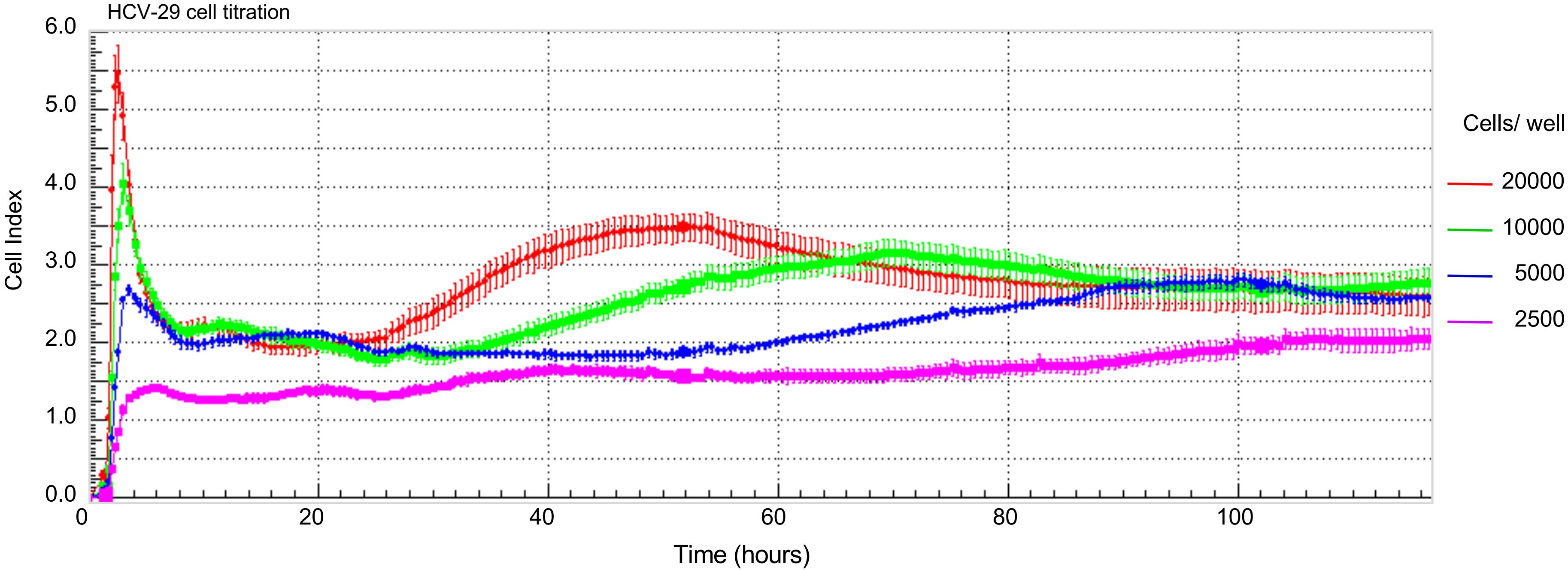

Figure S1. Cell titration assay for human urothelial cells HCV29. In order to analyze the real time cell proliferation kinetics for HCV29 cells, a cell titration assay seeding 2,500, 5,000, 10,000 and 20,000 cells per well was performed. Unprocessed data showing the cell index over time for each tested cell number per well as indicated are presented as displayed by RTCA Software 1.2 (ACEA). All curves represent the averages of three technical replicates for the experiment and standard deviations bars are shown at each data point.

Supplementary Figure S2

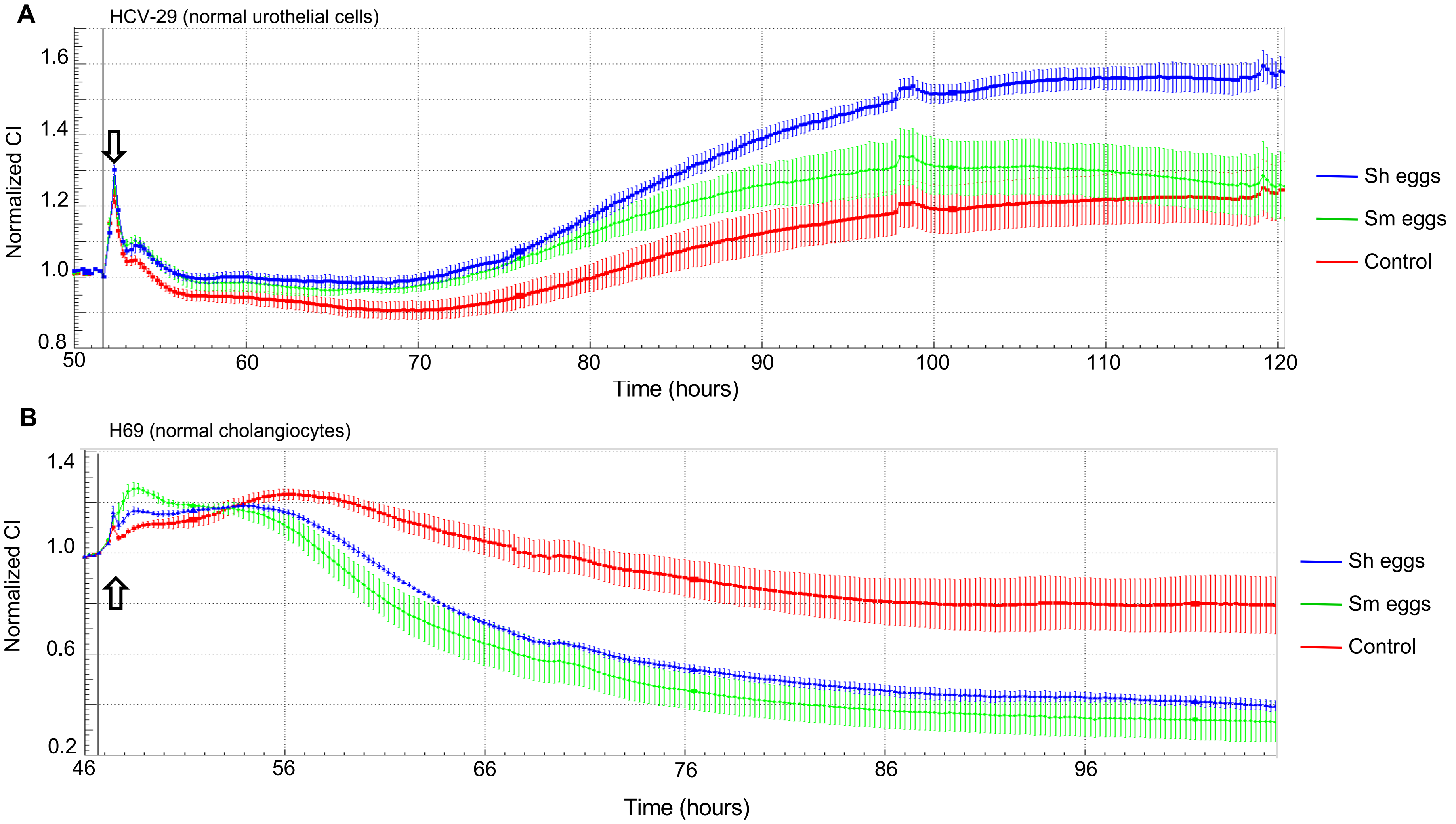

Figure S2. Cell growth measured in real time using the xCELLigence platform of cells co-cultured with schistosome eggs. Unprocessed data of representative experiments showing the normalized cell index (Normailzed CI) over time for HCV29 (Panel A) or H69 (B) cells co-cultured with *S. mansoni* eggs, *S. haematobium* eggs, or control cells co-cultured without eggs as indicated, are presented as displayed by RTCA Software 1.2 (ACEA). The normalization time is shown as a vertical black line, and the arrow indicated when eggs were added to the culture. All curves represent the averages of three technical replicates for the experiment and bar indicate the standard deviation at each data point.

Supplementary Figure S3

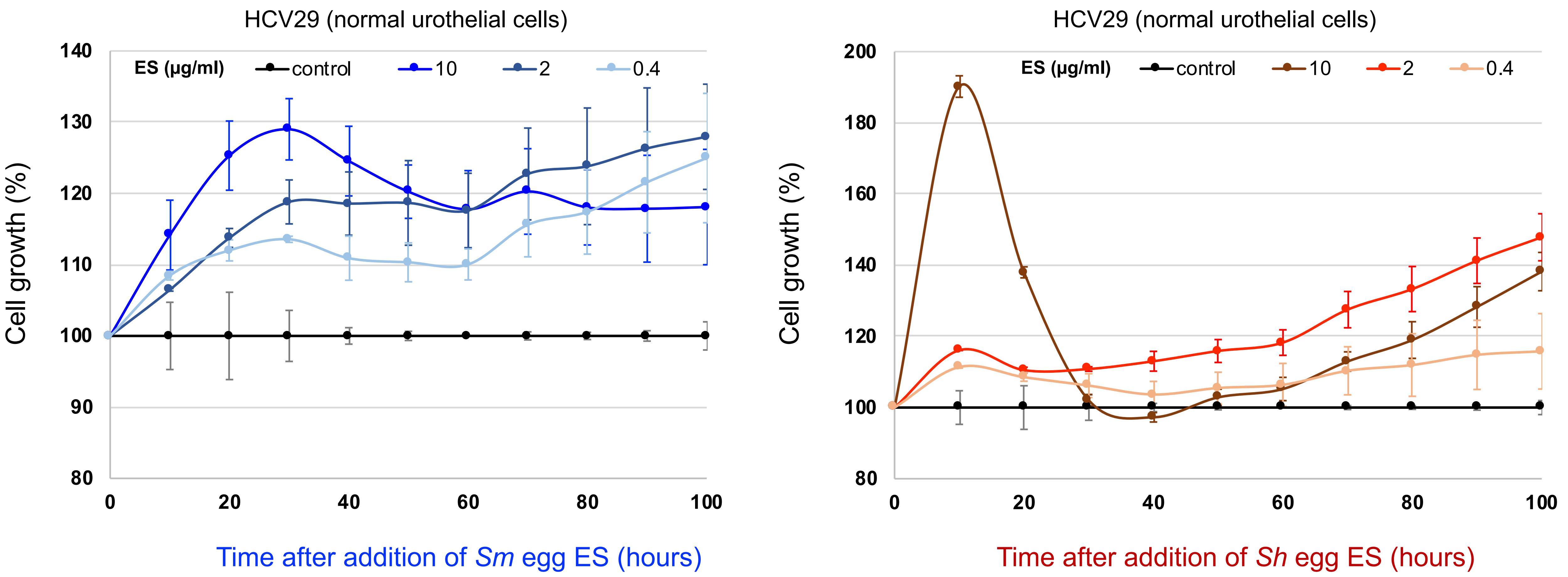

Figure S3. Cell growth measured in real time using the xCELLigence platform of cells co-cultured with schistosome egg excretory-secretory products (ES). HCV29 cells cultured in the presence of indicated concentrations of *S. mansoni* egg ES (left panel) or *S. haematobium* egg ES (right panel) over time after the addition of ES. Cell growth was expressed as percentage of the Normalized Cell Index of cells cultured with ES compared with control cells cultured in 1/20 diluted media without ES (control cell growth rate = 100%). All curves represent the averages of two technical replicates for the experiment and standard deviation bars are shown at each data point. *Sm* egg ES: *S. mansoni* egg excretory-secretory products, *Sh* egg ES: *S. haematobium* egg excretory-secretory products.

Supplementary Figure S4

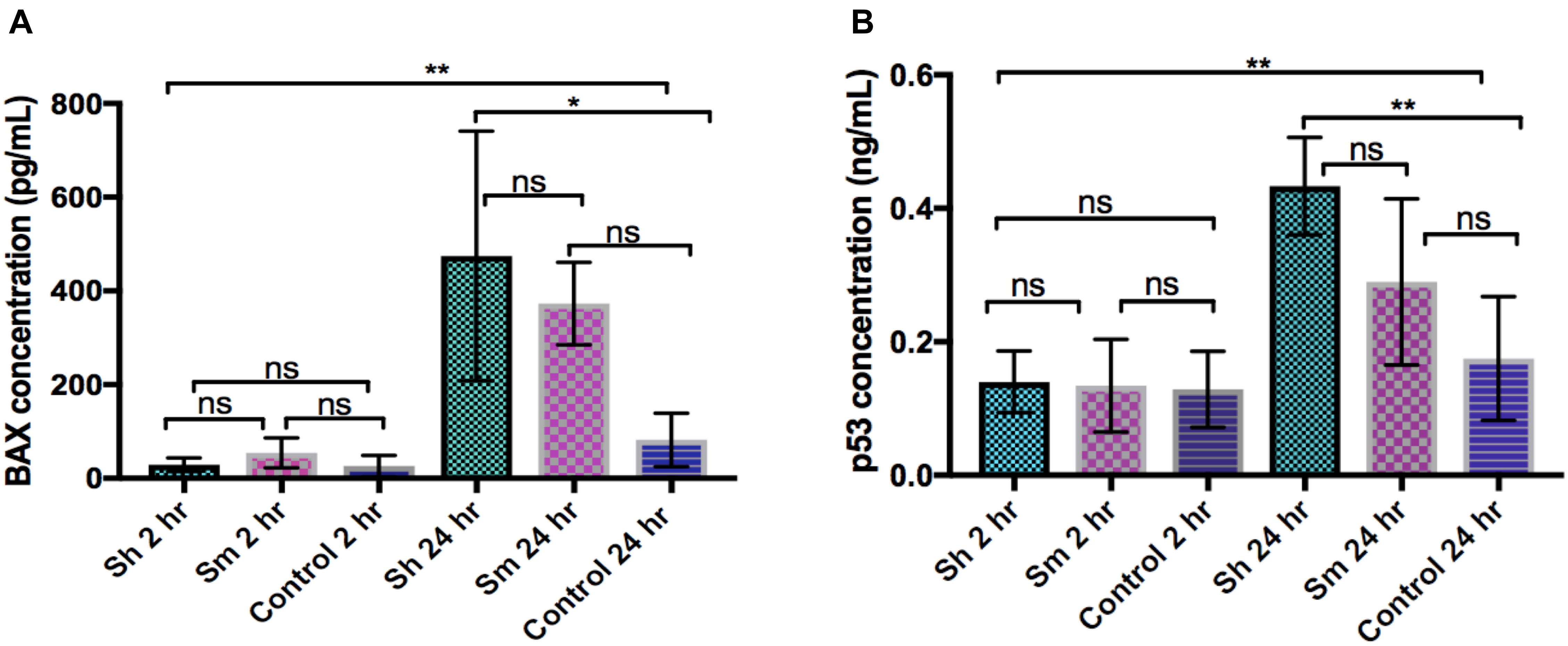

Figure S4. Changes in the concentration of selected human proteins induced by co-culturing schistosome eggs and human urothelial cells for 2 or 24 hours. Concentrations of the human BAX (panel A) or P53 (panel B) proteins measured by ELISA assays in the indicated experimental conditions. Sh: *Schistosoma haematobium* eggs, Sm: *Schistosoma mansoni* eggs. * indicates p≦0.05, ** indicates p≦0.01, ns indicates no significant difference.
